# Candesartan preserves aortic structure and function in cisplatin-treated rats by upregulating SIRT1/Nrf2/HO-1 signaling and suppressing oxidative stress, TLR-4/NF-κB signaling, and necroptosis

**DOI:** 10.3389/fphar.2025.1678921

**Published:** 2025-10-08

**Authors:** Ayman M. Mahmoud, Sulaiman M. Alnasser, Omnia A. M. Abd El-Ghafar, Reem S. Alruhaimi, Hanan S. Althagafy, Ahmed M. Atwa, Emad H. M. Hassanein

**Affiliations:** ^1^ Department of Life Sciences, Faculty of Science and Engineering, Manchester Metropolitan University, Manchester, United Kingdom; ^2^ Department of Pharmacology and Toxicology, College of Pharmacy, Qassim University, Buraydah, Saudi Arabia; ^3^ Department of Pharmacology and Toxicology, Faculty of Pharmacy, Nahda University, Beni-Suef, Egypt; ^4^ Department of Biology, College of Science, Princess Nourah bint Abdulrahman University, Riyadh, Saudi Arabia; ^5^ Division of Biochemistry, Department of Biological Sciences, Faculty of Science, University of Jeddah, Jeddah, Saudi Arabia; ^6^ Department of Pharmacology and Toxicology, Faculty of Pharmacy, Egyptian Russian University, Cairo, Egypt; ^7^ Department of Pharmacology and Toxicology, Faculty of Pharmacy, Al-Azhar University-Assiut, Assiut, Egypt

**Keywords:** candesartan, cisplatin, vascular injury, oxidative stress, inflammation

## Abstract

**Background:**

Cisplatin (CIS) is widely used in the treatment of several tumors. However, its use is associated with toxicity that contributes to long-term cardiovascular complications in cancer survivors. This study investigated whether the angiotensin II receptor blocker candesartan (CAN) could protect against CIS-induced aortic injury in rats.

**Methods:**

Rats received CAN (5 mg/kg, oral) for 10 days, with a single intraperitoneal dose of CIS (7 mg/kg) administered on day 7.

**Results:**

Histopathological analysis revealed that CIS induced extensive aortic damage, including endothelial disruption, elastic fiber fragmentation, thrombi, and medial calcification, which were significantly alleviated by CAN. CIS-induced oxidative stress was evidenced by elevated lipid peroxidation, myeloperoxidase (MPO) activity, and suppressed antioxidant defenses, while inflammatory activation was marked by upregulation of TLR-4, NF-κB, iNOS, and pro-inflammatory cytokines. CAN treatment reversed these alterations and restored redox balance and anti-inflammatory cytokine IL-10 levels. CAN enhanced SIRT1/Nrf2/HO-1 signaling and suppressed necroptosis-associated proteins (RIP1, RIP3, MLKL, and caspase-8). Molecular docking supported direct interactions between CAN and SIRT1, Keap1, and HO-1. Additionally, CAN corrected the CIS-induced imbalance in the renin-angiotensin system by decreasing angiotensin (Ang) II and increasing Ang-(1–7), and preserved endothelium-dependent vasorelaxation.

**Conclusion:**

These findings suggest that CAN protects against CIS-induced vascular injury through coordinated suppression of oxidative stress, inflammation, and necroptosis, alongside upregulation of SIRT1/Nrf2/HO-1 signaling and restoration of vascular function. CAN may represent a promising vascular-protective strategy in patients undergoing CIS chemotherapy.

## 1 Introduction

Cardiovascular diseases (CVDs) pose a significant global health burden, particularly in cancer patients where both the disease and its treatments elevate cardiovascular risk. Cancer patients exhibit a 55% increased cardiovascular mortality compared to the general population (Ng et al., 2024). For certain cancers, CVD can account for up to half of patient deaths and cancer survivors also face elevated risks of heart failure and stroke (Armstrong et al., 2013; Willems et al., 2022; Ng et al., 2024). While chemotherapy-induced cardiomyopathy has received considerable attention, drug-induced vascular toxicity represents a pervasive yet comparatively understudied dimension of this problem. Despite this, vascular injury is a common adverse effect of anticancer chemotherapies, often due to endothelial dysfunction (ED) (Szczepaniak et al., 2022; Terwoord et al., 2022). Cardiovascular adverse drug reactions contribute to a notable percentage of drug market withdrawals (Varga et al., 2015; Onakpoya et al., 2016; Seal et al., 2024). Chemotherapy increases susceptibility to vascular damage, and causes superior mesenteric artery embolism, coronary vasospasm, acute ventricular and aortic thrombosis, and other manifestations (Morlese et al., 2007; Fernandes et al., 2011; Sasaki et al., 2020; Aoki et al., 2023; Borregón et al., 2025). Direct chemotherapy-induced vascular injury is poorly characterized compared to other side effects. Vascular complications, encompassing ED, accelerated atherosclerosis, thromboembolism, and hypertension, contribute significantly to morbidity (Mehta et al., 2018; Herrmann, 2020; Terwoord et al., 2022) but remain less systematically characterized than direct myocardial injury in cancer patients. This knowledge gap demands urgent attention given the expanding population of cancer survivors living with latent cardiovascular complications.

Cisplatin (CIS), a platinum-based chemotherapeutic agent, is widely used for various cancers, including ovarian, testicular, and lung tumors (Brown et al., 2019). Its efficacy stems from DNA damage via platinum-DNA adducts, which distort the DNA helix, inhibiting replication and transcription, leading to apoptosis in rapidly dividing cancer cells (Brown et al., 2019; Ghosh, 2019). This mechanism has proven transformative, particularly for malignancies like testicular cancer, achieving high cure rates for localized disease. However, this therapeutic success is counterbalanced by a significant burden of off-target toxicities, including dose-limiting nephrotoxicity, neurotoxicity, ototoxicity, and increasingly recognized cardiovascular complications (Tsang et al., 2009; Amptoulach and Tsavaris, 2011; Madeddu et al., 2016). CIS-induced vascular toxicity manifests clinically as hypertension, Raynaud’s phenomenon, thromboembolic events, and accelerated coronary artery disease, affecting a substantial proportion of treated patients (Cameron et al., 2016). Critically, preclinical and clinical evidence indicates that vascular ED can manifest early, often at relatively low cumulative doses, potentially preceding overt cardiac systolic dysfunction (Herradón et al., 2017; Cameron et al., 2020). This early vascular vulnerability underscores the long-term cardiovascular risks faced by CIS survivors, persisting years after treatment cessation.

CIS-induced toxicity is linked to oxidative stress and inflammation. CIS provokes excessive generation of reactive oxygen species (ROS) driven by mitochondrial dysfunction, NADPH oxidase activation, and depletion of endogenous antioxidants, leading to oxidative damage (Brozovic et al., 2010; Rachma et al., 2025). This ROS surge induces macromolecular damage through lipid peroxidation (LPO), protein carbonylation, and DNA oxidation, directly impairing cell function. Critically, ROS act as potent second messengers to activate pro-inflammatory cascades (Asehnoune et al., 2004). Central to this inflammatory response is the toll-like receptor 4 (TLR-4)/nuclear factor-kappaB (NF-κB) axis. CIS promotes TLR-4 activation, triggering downstream signaling that culminates in NF-κB nuclear translocation (Asehnoune et al., 2004). NF-κB then orchestrates the transcription of key pro-inflammatory cytokines which perpetuate a state of endothelial activation, enhance leukocyte adhesion, and sustain vascular inflammation, establishing a self-perpetuating injury loop (Ramesh and Reeves, 2002; Pober and Sessa, 2007). Therefore, attenuation of oxidative and inflammatory responses promoted by CIS could be effective in preventing ED and aortic injury. In this context, activation of sirtuin 1 (SIRT1) and nuclear factor erythroid 2-related factor 2 (Nrf2) represents a pivotal cellular defense strategy against oxidative stress and inflammation (Yang et al., 2022). SIRT1, a NAD^+^-dependent deacetylase, modulates multiple redox-sensitive transcription factors, including NF-κB, thereby suppressing inflammatory gene expression and enhancing cellular resilience (Haigis and Guarente, 2006; Yang et al., 2022). Concurrently, Nrf2 is a master regulator of antioxidant responses; upon activation, it translocates to the nucleus and upregulates genes encoding antioxidant enzymes such as heme oxygenase 1 (HO-1) and glutamate-cysteine ligase catalytic subunit (GCLC), restoring redox homeostasis (Satta et al., 2017). Therefore, pharmacological upregulation of SIRT1 and Nrf2 offers an effective strategy to counteract vascular oxidative damage and inflammatory signaling triggered by CIS, ultimately preserving aortic structure and function.

Candesartan (CAN), an angiotensin (Ang) II receptor blocker (ARB), treats hypertension and heart failure by blocking the AT1 receptor, inhibiting Ang II’s vasoconstrictive, pro-inflammatory, and pro-fibrotic effects (Sever, 1997; Zhao et al., 2019). Beyond blood pressure regulation, CAN exhibits pleiotropic properties, including antioxidant and anti-inflammatory effects. Preclinical models have shown CAN mitigates oxidative and inflammatory injury in experimental disease models. It suppresses chronic renal inflammation by inhibiting redox-sensitive NF-κB pathways, independent of AT1 receptor blockade (Chen et al., 2008). In a rat model of CIS-induced lung injury, we have previously revealed that CAN ameliorated tissue damage by suppressing inflammation and oxidative stress and enhancing antioxidant defenses (Atwa et al., 2022). It exerted beneficial effects against acute myocardial infarction (AMI) (Lin et al., 2015), renal inflammation (Chen et al., 2008), and stroke-induced neuronal damage (Qie et al., 2020). However, its potential to specifically protect against CIS-induced vascular injury, particularly through modulating inflammation, oxidative stress and necroptosis while enhancing endogenous cytoprotective pathways like SIRT1/Nrf2/HO-1, remains largely unexplored. This study aims to investigate the effects of CAN against CIS-induced vascular toxicity in rats. We hypothesize that CAN mitigates CIS-induced thoracic aorta injury by suppressing oxidative stress, TLR-4/NF-κB signaling, pro-inflammatory cytokines, and necroptosis, while upregulating SIRT1/Nrf2/HO-1 signaling. The thoracic aorta is a central elastic artery that plays a pivotal role in maintaining cardiovascular homeostasis by regulating arterial elasticity, blood pressure, and steady blood flow to vital organs (Laurent and Boutouyrie, 2015). ED and structural or functional alterations of the aorta contribute significantly to the pathogenesis of thrombosis, hypertension, atherosclerosis, heart failure, and stroke (Laurent and Boutouyrie, 2015; Khorana et al., 2022) and acute aortic thrombosis in response to CIS has been reported (Fernandes et al., 2011; Borregón et al., 2025). Cancer patients on chemotherapy are at an elevated risk of developing vascular injury and subsequent cardiovascular complications (Herrmann, 2020). Therefore, studying the thoracic aorta provides valuable insights into vascular dysfunction associated with chemotherapy and the potential protective effects of therapeutic interventions.

## 2 Materials and methods

### 2.1 Animals and treatments

Twenty-four adult male Wistar rats (180–210 g) were housed under standard laboratory conditions (temperature 22 °C–24 °C, 12 h light/dark cycle, and relative humidity of 50%–60%) with unrestricted access to food and water. The experimental protocol was approved by the Institutional Animal Ethics Committee of Al-Azhar University (Approval No. ZA-AS/PH/15/C/2022) and adhered to international guidelines for animal care.

Rats were randomly allocated into four groups (*n* = 6):

Group I (Control): received daily oral administration of vehicle (saline) for 10 days and an intraperitoneal (i.p.) saline injection on day 7.
Group II (CAN): received 5 mg/kg CAN (Thakur et al., 2015; Atwa et al., 2022) orally for 10 consecutive days and a saline injection (i.p.) on day 7.
Group III (CIS): administered saline orally for 10 days, with a single dose of 7 mg/kg CIS (Aladaileh et al., 2021; Atwa et al., 2022) (i.p.) given on day 7.
Group IV (CAN + CIS): treated with 5 mg/kg CAN orally for 10 days and received CIS (7 mg/kg, i. p.) on day 7.

CAN and CIS were supplied by AstraZeneca (Egypt) and Sigma (USA), respectively. On day 11, rats were anesthetized using ketamine/xylazine and sacrificed. The thoracic aorta was carefully excised, rinsed in cold PBS, and processed for downstream analysis. Aortic tissues were either fixed in 10% neutral-buffered formalin (NBF) for histological and immunohistochemical studies, stored at −80 °C for molecular assessments, or freshly processed for functional vascular studies.

### 2.2 Histology and immunohistochemistry (IHC)

Formalin-fixed aortic segments were paraffin-embedded, sectioned at 5 μm, and stained with hematoxylin and eosin (H&E) for general histopathological evaluation under light microscopy. For IHC, sections underwent deparaffinization, rehydration, and antigen retrieval in citrate buffer (50 mM, pH 6.8). Endogenous peroxidase was blocked using 0.3% hydrogen peroxide, followed by incubation with 1% bovine serum albumin (BSA). Primary antibodies targeting SIRT1, Nrf2, NF-κB p65, TLR-4, and inducible nitric oxide synthase (iNOS) (Biospes, China) were applied overnight at 4 °C. After washing, appropriate HRP-conjugated secondary antibodies were used, and staining was visualized with DAB and hematoxylin was employed for counterstaining. Digital images were analyzed using ImageJ software (NIH, USA) for quantification.

### 2.3 Biochemical analyses

Aortic tissues were homogenized (10% w/v) in Tris-HCl buffer (pH 7.4), then centrifuged at 4 °C. The supernatant was used for biochemical assays and ELISA. LPO, GSH, and nitrite levels and activities of superoxide dismutase (SOD), glutathione peroxidase (GPx) and glutathione-s-transferase (GST) were assayed using BioDiagnostic (Egypt) kits, following the manufacturer’s instructions. Activities of myeloperoxidase (MPO) and HO-1 were assayed following the methods of Krawisz et al. (1984) and Abraham et al. (1985), respectively. ELISA kits (Elabscience, China) were used to quantify tumor necrosis factor (TNF)-α, interleukin (IL)-1β, IL-10, Ang II, and Ang-(1–7) according to the manufacturer’s protocols.

### 2.4 Quantitative real-time PCR (qRT-PCR)

Total RNA was extracted from aortic samples using Trizol reagent (Invitogrn) and quantified spectrophotometrically. cDNA was synthesized using a high-capacity cDNA synthesis kit (Thermo Fisher), and qPCR was carried out using SYBR Green Master Mix (Thermo Fisher) on a real-time thermal cycler. Specific primers targeting HO-1, GCLC, kelch like ECH associated protein 1 (Keap1), and GAPDH were used (Table 1). The results were analyzed using the 2^−ΔΔCT^ method (Livak and Schmittgen, 2001).

**TABLE 1:** Primers used for qRT-PCR.

Gene	Forward primer (5′–3′)	Reverse primer (5′–3′)
Nrf2	TTG​TAG​ATG​ACC​ATG​AGT​CGC	TGT​CCT​GCT​GTA​TGC​TGC​TT
GCLC	GTT​GTT​ACT​GAA​TGG​CGG​CG	CGG​CGT​TTC​CTC​ATG​TTG​TC
HO-1	GTA​AAT​GCA​GTG​TTG​GCC​CC	ATG​TGC​CAG​GCA​TCT​CCT​TC
Keap1	TCA​GCT​AGA​GGC​GTA​CTG​GA	TTC​GGT​TAC​CAT​CCT​GCG​AG
GAPDH	TGC​TGG​TGC​TGA​GTA​TGT​CG	TTGAGAGCAATGCCAGCC

### 2.5 Western blotting

Protein expression of necroptosis-related markers was assessed using Western blotting. Frozen aortic tissues were homogenized in RIPA buffer supplemented with a protease inhibitor cocktail, followed by centrifugation at 12,000 rpm for 15 min at 4 °C. The supernatant was collected, and total protein concentration was determined using the Bradford assay. Equal amounts of protein (50 µg) were separated on SDS-polyacrylamide gels and transferred onto PVDF membranes. After blocking with 5% BSA in Tris-buffered saline containing 0.1% Tween-20 (TBST), membranes were incubated overnight at 4 °C with primary antibodies against receptor-interacting protein 1 (RIP1), RIP3, mixed lineage kinase domain like (MLKL), caspase-8 and β-actin. Antibodies were obtained from Santa Cruz Biotechnology (United States) and Biospes (China). After washing, membranes were probed with species-specific secondary antibodies for 1 h at room temperature. Bands were visualized using a colorimetric detection system (BCIP/NBT), and the intensity of protein bands was quantified using ImageJ software (NIH, United States). The results were normalized to β-actin expression and expressed as relative protein levels.

### 2.6 Vascular reactivity assessment

Thoracic aortae were excised and cleaned of connective tissue, then cut into rings of approximately 3 mm in length. Each ring was mounted in a 5 mL organ bath chamber filled with Krebs-Henseleit solution (mM: NaCl, 118.0; KCl, 4.7; CaCl_2_, 2.5; KH_2_PO_4_, 1.2; MgSO_4_, 1.2; NaHCO_3_, 25.0; and glucose, 10.0), maintained at pH 7.4, 37 °C, and continuously aerated with a gas mixture of 95% O_2_ and 5% CO_2_. Rings were allowed to equilibrate for 60 min under a resting tension of 1 g, with solution replacement every 15 min (Ameer et al., 2015; Panthiya et al., 2019). After equilibration, endothelial integrity was verified by relaxation to acetylcholine (ACh, 10^−6^ M) following submaximal pre-contraction with phenylephrine (PE, 10^−6^ M). Endothelium-dependent relaxation responses were obtained using cumulative concentrations of ACh added to the bath after steady contraction was achieved. Isometric tension was recorded using a force transducer system (ADInstruments, Australia).

### 2.7 Molecular docking

To predict the binding affinity of CAN to SIRT1, Keap1, and HO-1, protein structures were retrieved from the RCSB Protein Data Bank (PDB IDs: 5BTR, 5CGJ, and 1DVE, respectively) and prepared using AutoDock Tools (v1.5.6). Docking was performed using AutoDock Vina integrated in PyRx (v0.8) (Dallakyan and Olson, 2015), and ligand-receptor interactions were visualized and analyzed using PyMOL (v2.3.2) and LigPlot+ (v2.2.8) (Wallace et al., 1995).

### 2.8 Statistical analysis

Data were presented as mean ± standard error (SEM). Comparisons among groups were made using one-way ANOVA followed by Tukey’s *post hoc* test. Statistical significance was considered at P < 0.05. GraphPad Prism version 8.0 was used for all analyses.

## 3 Results

### 3.1 CAN prevents CIS-induced histopathological alterations in rat aorta

**FIGURE 1:**
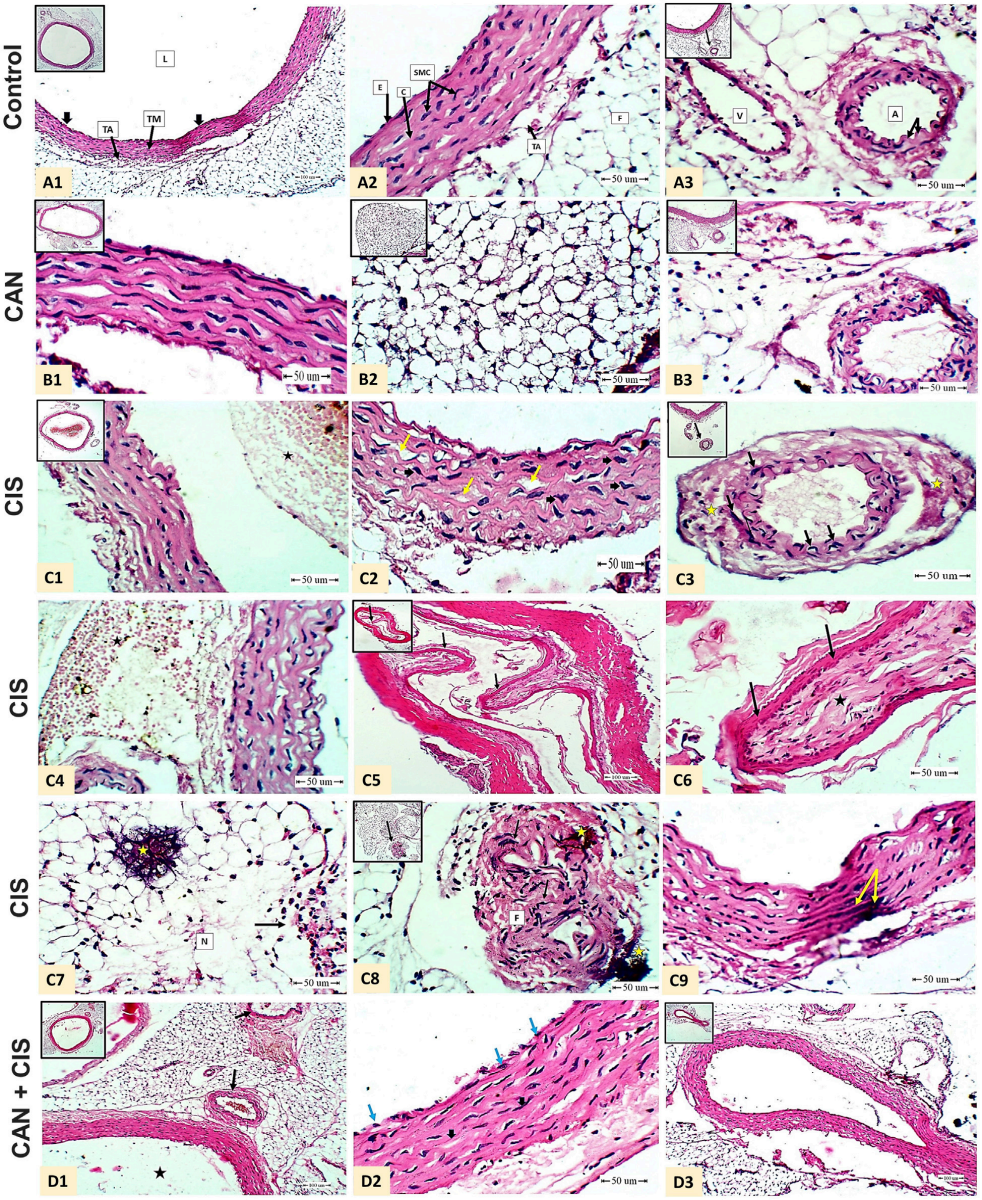
CAN mitigated CIS-induced aortic damage in rats. Representative H&E-stained aortic sections demonstrate structural integrity in Control rats **(A1-A3)** A1: shows normal tunica intima (endothelial cells, thick black arrow), tunica media (TM), tunica adventitia (TA), and lumen (L) (Scale bar = 100 µm); A2: details endothelial cells (E), smooth muscle cells (SMC), collagen (C), TA, and adipose tissue (F) (Scale bar = 50 µm); and A3: displays periaortic adipose tissue containing a normal artery (elastic lamina, black arrow) and vein (V) (Scale bar = 50 µm). CAN-alone rats **(B1-B3)** exhibit normal aortic structure identical to controls. CIS-administered rats **(C1-C9)** exhibit severe pathology. C1: shows white thrombus (platelets and white blood cells, star) near by the wall of the aorta (Scale bar = 50 µm); C2: shows marked corrugation and widening of elastic fibers (yellow arrow) and enlargement mis-shaped smooth muscle cells nuclei (thick black arrow) in the tunica media of the aorta (Scale bar = 50 µm); C3: shows artery associated with the aorta beneath the adipose tissue showed mis-shaped elongated nuclei of the smooth muscle cells in the tunica media (black arrow) and early fibrinoid necrosis (star) (Scale bar = 50 µm); C4: shows massive area of extra-vasated red blood cells (hemorrhage, star) beneath the tunica adventitia (Scale bar = 50 µm); C5: shows myxomatus polyp arising from the tunica media of an associated artery of the aorta (branch) toward the lumen (black arrow) (Scale bar = 100 µm); C6: higher magnification of C5 shows connective tissue core(star) covered by epithelium (black arrow) (Scale bar = 50 µm); C7: shows area of fat necrosis (N) of the adipose tissue surrounding the aorta with calcification (yellow star) and inflammatory cellular reaction (black arrow) (Scale bar = 50 µm); C8: shows mis-shaped elongated nuclei (black arrow) and fibrinoid necrosis (F) and calcification (yellow star) (Scale bar = 50 µm); and C9: shows medial calcification (yellow arrow) (Scale bar = 50 µm). CIS + CAN rats **(D1-D3)** shows absence of the mural thrombus in the lumen (black star) and apparent normal arteries (black arrow) beneath the aorta in the adipose tissue (D1 - Scale bar = 100 µm); intact endothelial lining the tunica intima (blue arrow), wavy uniform bundles of elastic fibers, apparent normal smooth muscle cell nuclei and less widening (Thick arrow) (D2 - Scale bar = 50 µm); and normal periaortic artery without polyps (D3 -Scale bar = 100 µm).

Histopathological evaluation of aortic sections from the control group revealed normal vascular architecture. The tunica intima was lined by flat endothelial cells, the tunica media displayed organized smooth muscle cells interspersed with elastic fibers, and the tunica adventitia showed typical connective tissue and adipose components. The vascular lumen appeared wide and unobstructed, and associated arteries and veins were structurally intact (Figures 1A1–A3). CAN-treated rats displayed aortic histology similar to controls, with preserved endothelial lining, normal elastic lamellae, and smooth muscle cell morphology, indicating no structural abnormalities associated with the treatment (Figures 1B1–B3). In contrast, CIS-treated rats exhibited vascular damage revealed by the presence of thrombi composed of platelets and leukocytes near the aortic wall, marked corrugation and fragmentation of elastic fibers in the tunica media, and distorted, irregularly shaped nuclei of smooth muscle cells. Early signs of fibrinoid necrosis were evident. Additional features included extensive sub-adventitial hemorrhage, development of myxomatous polyps protruding from the media into the vascular lumen, adipose tissue necrosis with calcific deposits, and an intense inflammatory cellular infiltrate. Medial calcification was also frequently observed (Figures 1C1–C9). CAN markedly attenuated these pathological changes where the aortic sections showed preserved endothelial integrity, uniform elastic fibers, and well-organized smooth muscle cell nuclei. There was a noticeable absence of thrombi, myxomatous polyps, and calcific deposits, and the associated arteries appeared histologically normal (Figures 1D1–D3).

### 3.2 CAN alleviates CIS-induced oxidative stress in the aorta

**FIGURE 2:**
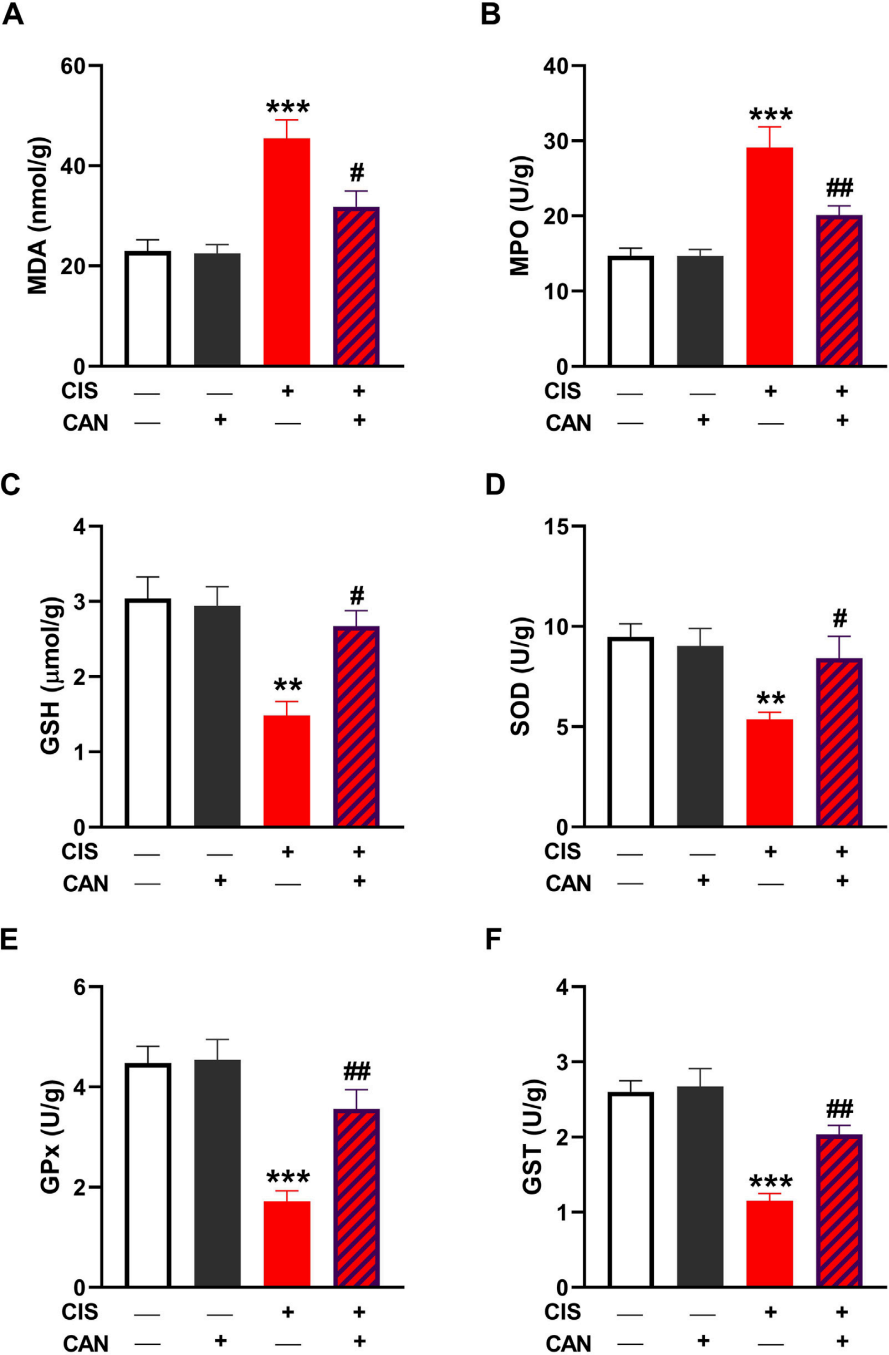
CAN alleviated CIS-induced oxidative stress in the aorta. CAN decreased MDA **(A)**, suppressed MPO **(B)**, increased GSH **(C)**, and enhanced SOD **(D)**, GPx **(E)**, and GST **(F)** in the aorta of CIS-administered rats. Data are mean ± SEM, (*n* = 6). **P < 0.01 and ***P < 0.001 vs. Control. #P < 0.05, ##P < 0.01, and ###P < 0.001 vs. CIS.

CIS administration resulted in pronounced oxidative injury in aortic tissue, as indicated by a significant increase in MDA (Figure 2A) and MPO (Figure 2B) compared to control rats (P < 0.001). Treatment with CAN notably mitigated this oxidative insult, significantly reducing both MDA and MPO (P < 0.05 and P < 0.01, respectively). Additionally, CIS markedly suppressed the key antioxidant defenses, including GSH, SOD, GPx, and GST (Figures 2C–F). CAN restored these antioxidant parameters, with significant improvements observed in GSH, SOD, GPx, and GST.

### 3.3 CAN suppresses CIS-induced activation of TLR-4/NF-κB pathway in aortic tissue

**FIGURE 3:**
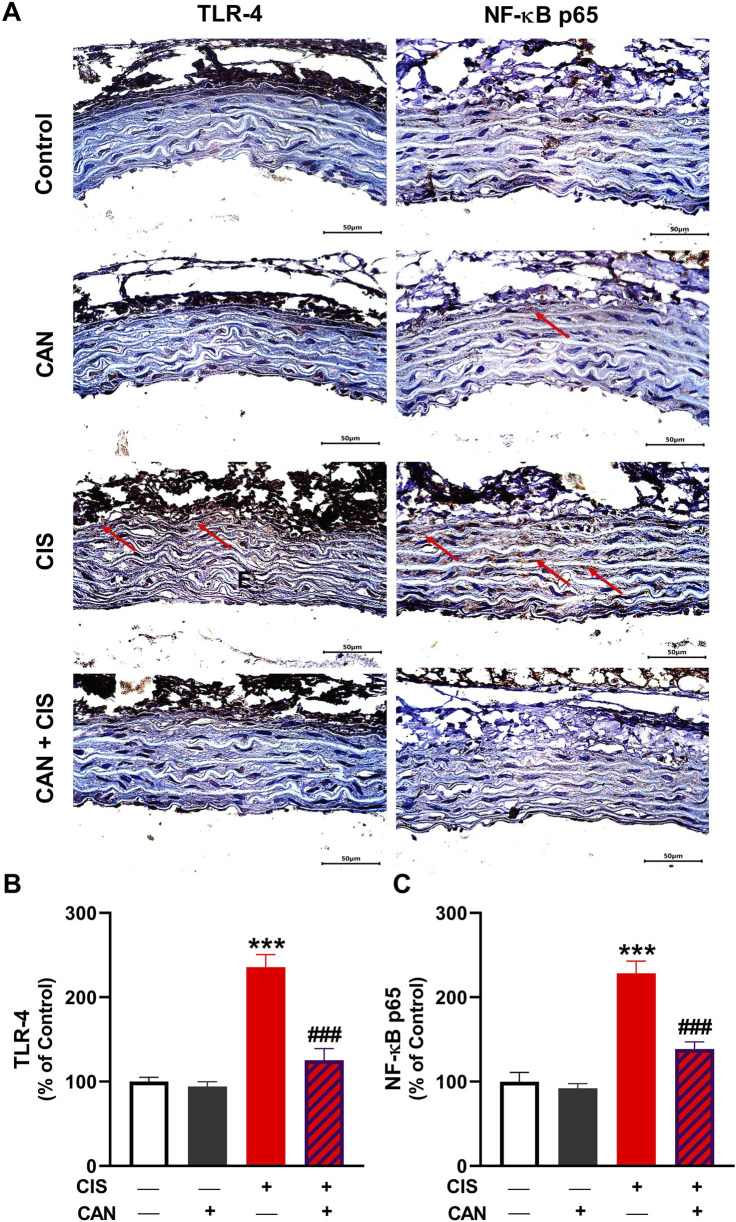
CAN downregulated TLR-4 **(A,B)** and NF-κB **(A,C)** in CIS-administered rat aorta. Data are mean ± SEM, (*n* = 6). ***P < 0.001 vs. Control and ###P < 0.001 vs. CIS.

**FIGURE 4:**
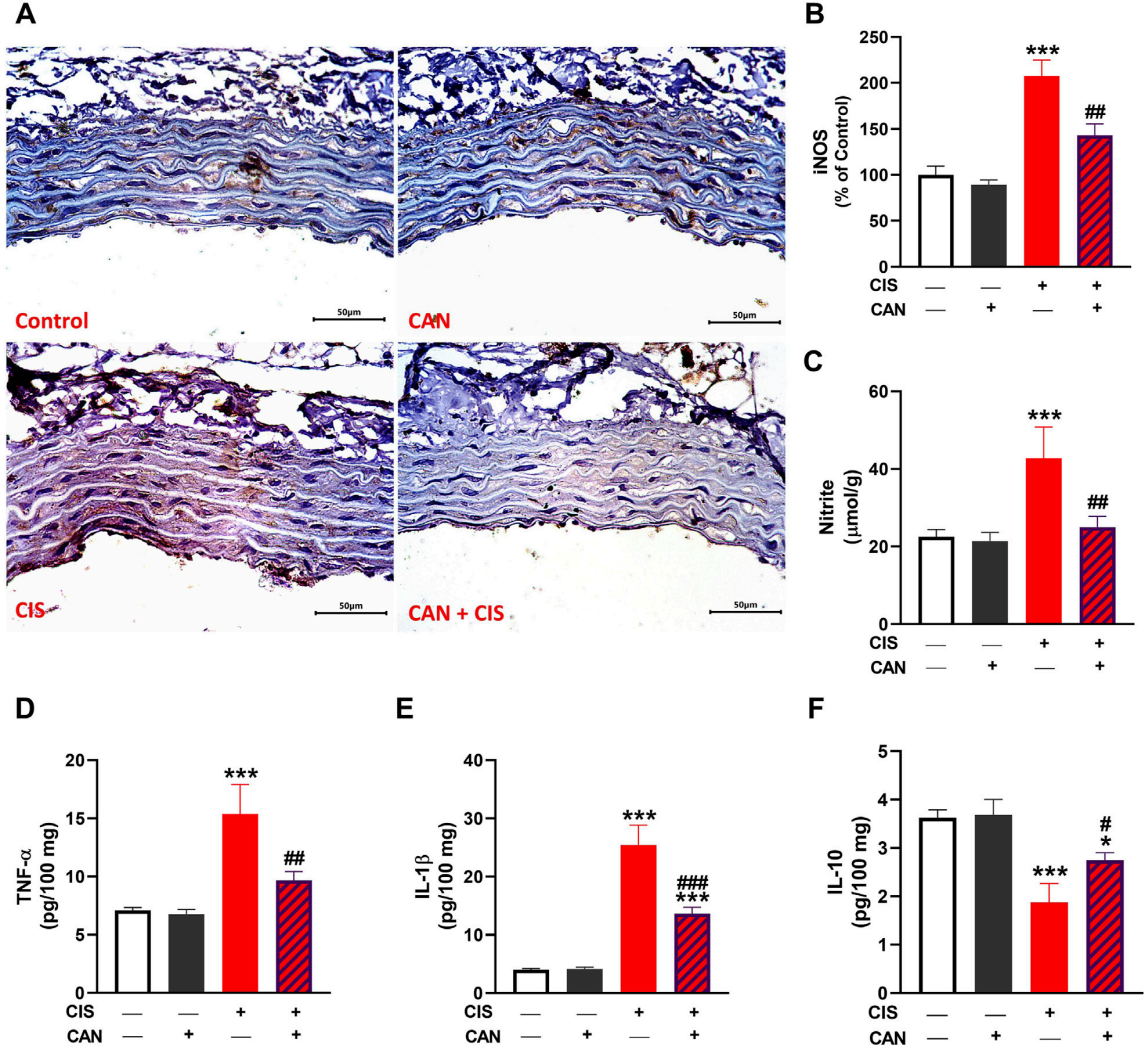
CAN alleviated CIS-induced aortic inflammation. CAN downregulated iNOS **(A,B)**, nitrite **(C)**, TNF-α **(D)** and IL-1β **(E)**,and increased IL-10 **(F)** in the aorta of CIS-administered rats. Data are mean ± SEM, (*n* = 6). *P < 0.05 and ***P < 0.001 vs. Control. #P < 0.05, ##P < 0.01, and ###P < 0.001 vs. CIS.

IHC and quantitative analysis revealed that CIS significantly upregulated TLR-4 and NF-κB p65 in aortic sections compared to the control group (P < 0.001; Figures 3A–C). CAN markedly attenuated this upregulation, as evidenced by the significant decrease in both TLR-4 and NF-κB p65 (P < 0.001). CIS significantly elevated iNOS expression and nitrite levels in the aorta, indicating increased nitrosative stress (P < 0.001; Figures 4A–C). CAN significantly suppressed both iNOS expression and nitrite production (P < 0.01). Moreover, CIS increased TNF-α and IL-1β (P < 0.001; Figures 4D,E), while simultaneously reducing IL-10 (P < 0.001; Figure 4F). CAN reversed these changes, significantly decreasing TNF-α and IL-1β levels (P < 0.01 and P < 0.001, respectively) and restoring IL-10 concentrations (P < 0.05).

### 3.4 CAN inhibits CIS-induced necroptosis in rat aorta

**FIGURE 5:**
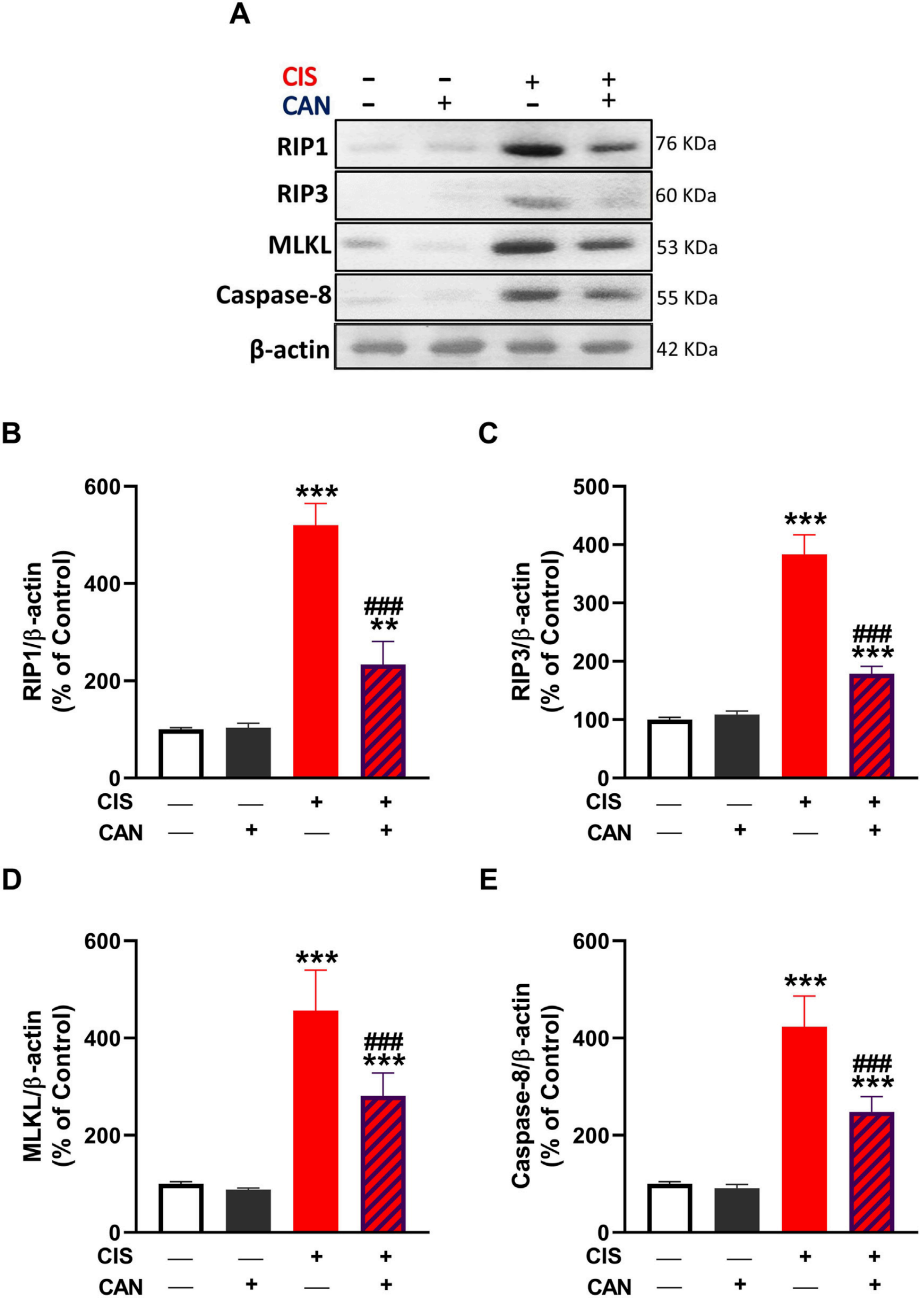
CAN attenuated CIS-induced necroptosis in rat aortic tissue. **(A)** Representative Western blot images showing protein expression levels of RIP1, RIP3, MLKL, and caspase-8. **(B–E)** Quantification of protein expression normalized to β-actin and expressed as a percentage of control for RIP1 **(B)**, RIP3 **(C)**, MLKL **(D)**, and caspase-8 **(E)**. Data are presented as mean ± SEM (*n* = 6). **P < 0.01 and ***P < 0.001 vs. Control. ###P < 0.001 vs. CIS.

CIS significantly enhanced the expression of key necroptotic markers, RIP1, RIP3, MLKL, and caspase-8 in aortic tissue as compared to the control rats (P < 0.001; Figures 5A–E). CAN substantially reduced the expression levels of RIP1, RIP3, MLKL, and caspase-8 (P < 0.001).

### 3.5 CAN upregulates aortic SIRT1/Nrf2/HO-1 signaling in CIS-treated rats

**FIGURE 6:**
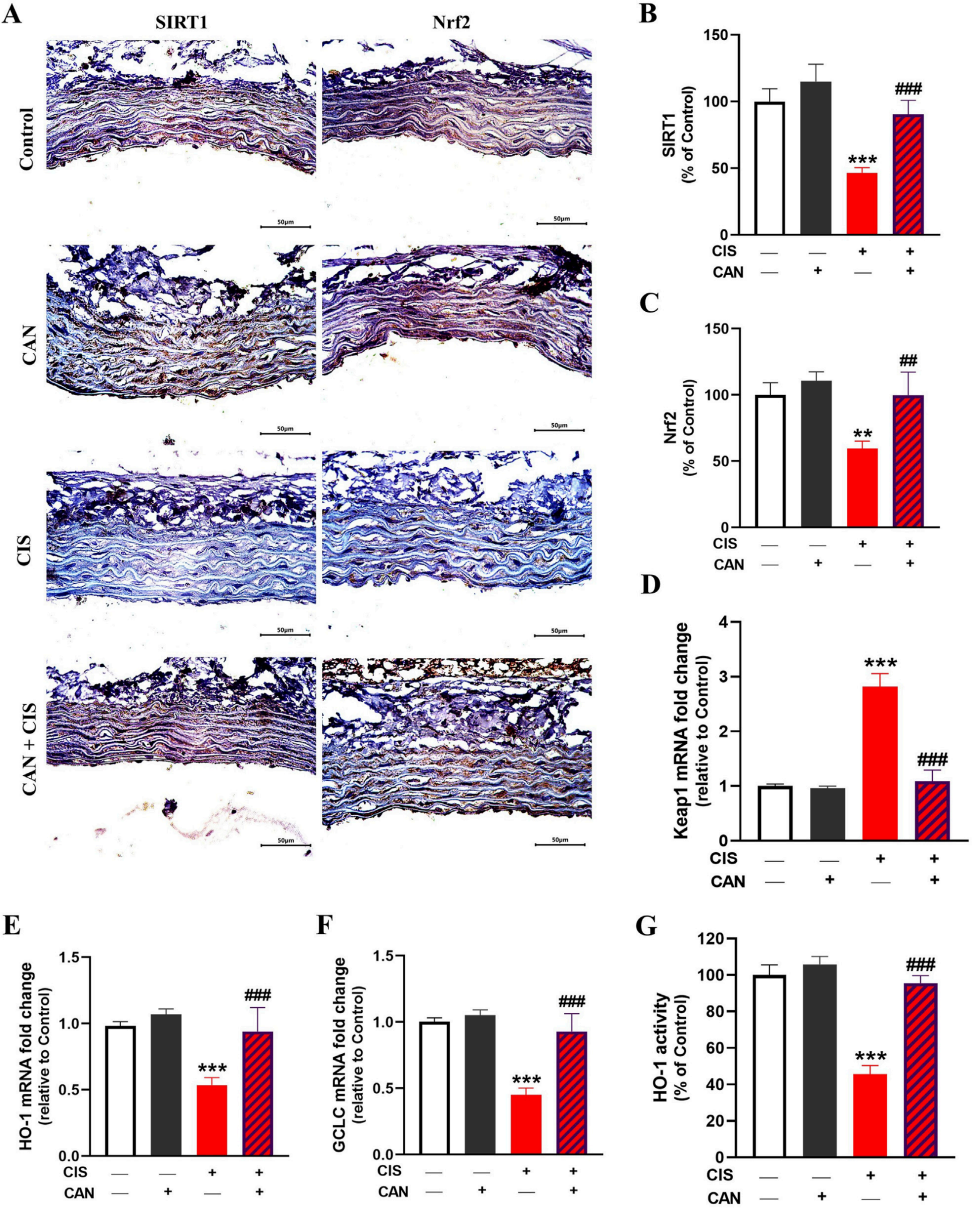
CAN upregulated SIRT1/Nrf2/HO-1 signaling in the aorta of CIS-administered rats. CAN upregulated SIRT1 **(A,B)**, Nrf2 **(A,C)**, decreased Keap1 mRNA **(D)**, increased HO-1 **(E)** and GCLC mRNA **(F)**, and enhanced HO-1 activity **(G)**. Data are mean ± SEM, (*n* = 6). **P < 0.01 and ***P < 0.001 vs. Control. ##P < 0.01, and ###P < 0.001 vs. CIS.

CIS administration resulted in a marked suppression of the SIRT1/Nrf2/HO-1 signaling in aortic tissue. IHC showed significantly reduced expression of both SIRT1 (P < 0.001) and Nrf2 (P < 0.01) in the CIS group compared to controls (Figures 6A–C). Consistently, the data revealed significant downregulation of HO-1 (Figure 6D) and GCLC (Figure 6E) mRNA levels, while biochemical assays demonstrated a corresponding decrease in HO-1 enzymatic activity (Figure 6F) (P < 0.001). CAN restored SIRT1 and Nrf2 and significantly upregulated HO-1 and GCLC transcripts, along with increased HO-1 enzymatic activity.

**FIGURE 7:**
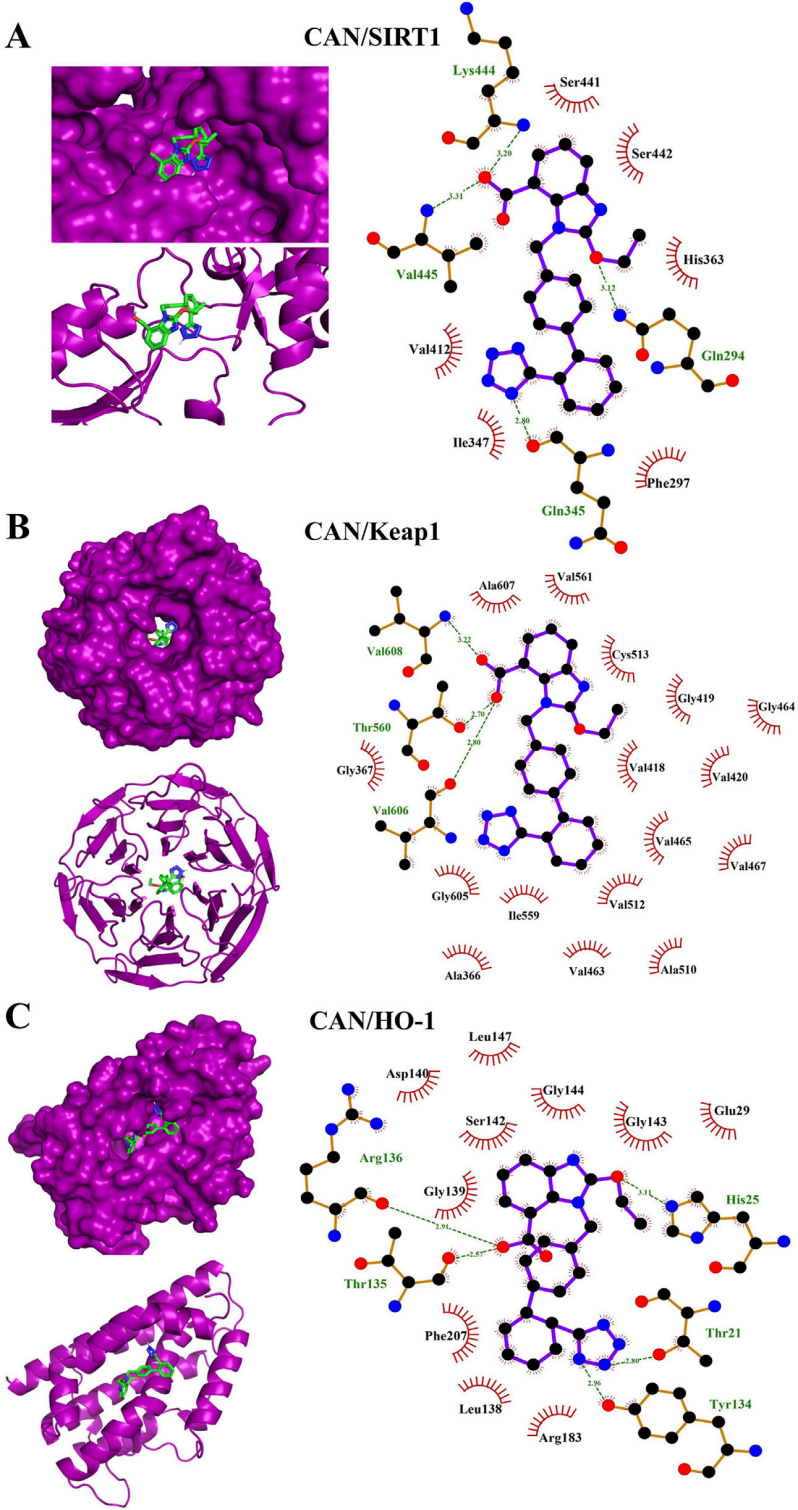
Molecular docking shows the binding affinity of CAN towards SIRT1 **(A)**, Keap1 **(B)**, and HO-1 **(C)**.

*In silico* simulations were performed to explore the potential interaction of CAN with SIRT1, Keap1, and HO-1 (Figures 7A–C). The interaction with SIRT1 was stabilized through 4 polar bonds, along with 6 hydrophobic contacts (Table 2). Similarly, CAN exhibited strong affinity for Keap1, engaging in hydrogen bonding with 3 residues and hydrophobic interactions with 16 residues (Table 2). Binding to HO-1 also involved a combination of hydrogen bonds and hydrophobic interactions with 5 and 10 amino acid residues, respectively (Table 2).

**TABLE 2:** Binding affinities and interaction of CAN with SIRT1, Keap1, and HO-1.

Protein	Lowest binding energy (kcal/mol)	Polar bonds	Hydrophobic interactions
SIRT1	−8.5	Lys444, Val445, Gln345, Gln294	Val412, Ile347, Ser441, Ser442, His363, Phe297
Keap1	−9.1	Val608, Val606, Thr560	Gly367, Ala607, Val561, Cys513, Gly605, Val512, Ile559, Ala366, Val418, Gly419, Val420, Val465, Val467, Val463, Ala510, Gly464
HO-1	−8.5	Arg136, Thr135, Thr21, Tyr134, His 25	Asp140, Leu147, Gly139, Ser142, Gly144, Phe207, Leu138, Arg183, Glu29, Gly143

### 3.6 CAN restores the balance of angiotensin peptides and preserves endothelium-dependent vasodilation impaired by CIS

**FIGURE 8:**
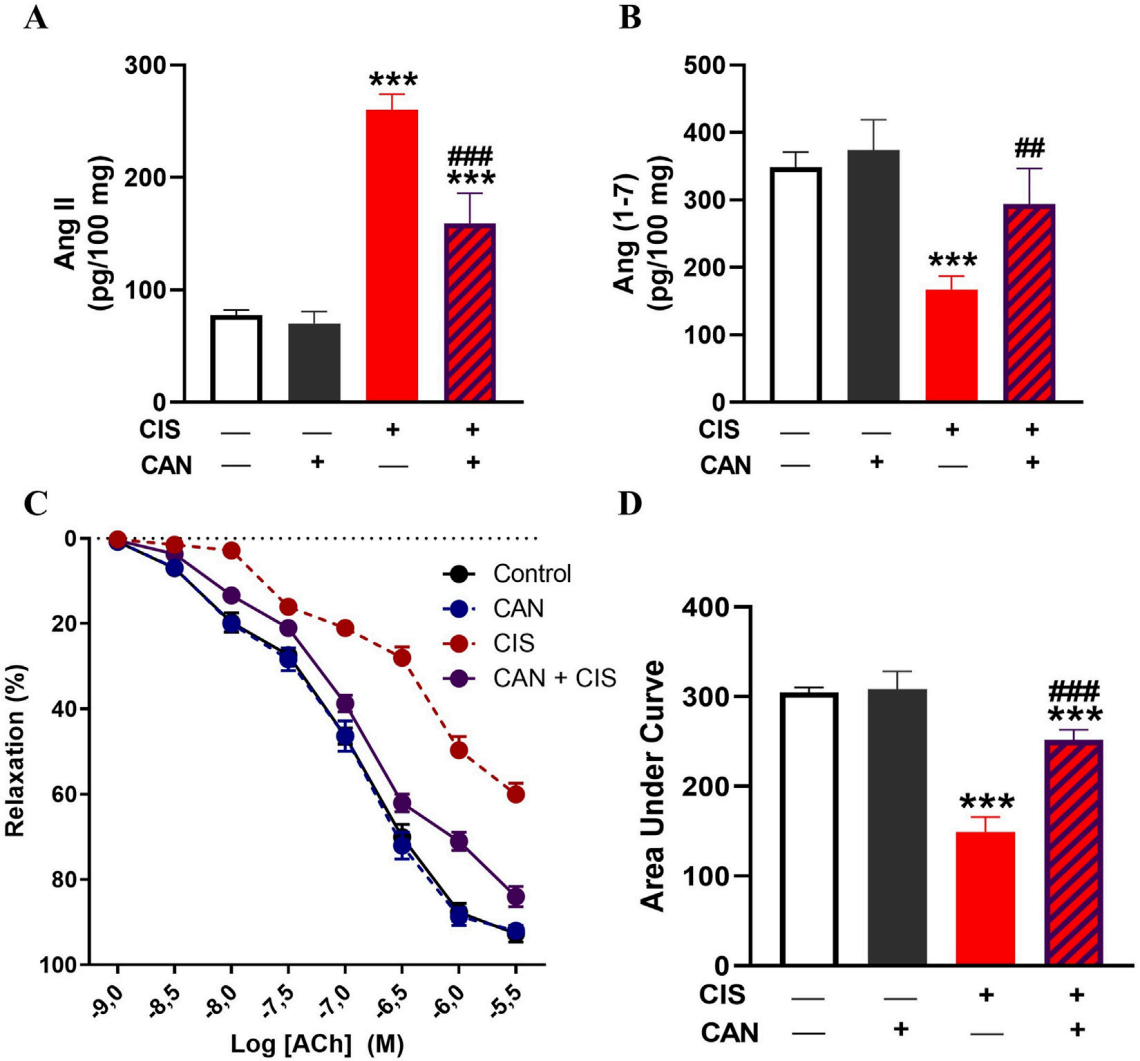
CAN decreased aortic Ang II **(A)**, increased Ang-(1–7) **(B)**, and improved endothelium dependent vasodilation **(C,D)** in CIS-administered rats. Data are mean ± SEM, (*n* = 6). ***P < 0.001 vs. Control. ##P < 0.01, and ###P < 0.001 vs. CIS.

CIS markedly increased Ang II (Figure 8A) and reduced Ang-(1–7) (Figure 8B) levels compared to controls (P < 0.001). CAN effectively alleviated these alterations, significantly lowering Ang II and restoring Ang-(1–7), suggesting that CAN can also promotes the protective, vasodilatory arm of the renin-angiotensin system (RAS). Vascular reactivity studies using aortic ring myography demonstrated that CIS significantly impaired endothelium-dependent relaxation responses to ACh (P < 0.001 vs. control; Figures 8–C–D). The maximal relaxation was markedly reduced, and the sensitivity to ACh was significantly attenuated in CIS-treated rats. In contrast, CAN effectively preserved endothelial function, as revealed by improvement in ACh-induced vasorelaxation (P < 0.001 vs. CIS), restoring both maximal response and vascular sensitivity toward near-control levels. The reported EC_50_ (and %E_max_) values are 1.18 × 10^−7^ M (95.64), 1.07 × 10^−7^ M (93.87), 1.34 × 10^−6^ M (68.11), and 1.36 × 10^−7^ M (84.01) for control, CAN, CIS and CAN + CIS groups, respectively.

## 4 Discussion

Despite growing recognition of drug-induced vascular toxicity, the precise mechanisms of CIS-induced vascular injury and effective interventions remain unclear. The present study provides compelling evidence for the multifaceted protective effects of CAN against CIS-induced vascular toxicity in rats. Our findings demonstrate that CIS administration inflicts significant structural and functional damage to the aorta, characterized by severe histopathological alterations, exacerbated oxidative stress, profound inflammatory responses, activation of necroptosis, and impaired antioxidant defense mechanisms. CAN consistently mitigated all these detrimental effects, underscoring its comprehensive vasculoprotective properties. These results collectively support our hypothesis that CAN exerts its beneficial actions by suppressing oxidative stress, inflammation, and necroptosis, while simultaneously upregulating key endogenous protective signaling pathways.

Histopathological examination revealed robust evidence of CIS-induced vascular injury. CIS caused severe endothelial damage, a critical initial step in vascular dysfunction (Feldman et al., 2024; Cameron et al., 2020; Pan et al., 2022). The presence of white thrombi indicated endothelial perturbation and a pro-thrombotic state (Badimon and Vilahur, 2014). Medial calcification, a hallmark of advanced vascular disease, suggested chronic vascular stress and dysregulation of calcium metabolism (Badimon and Vilahur, 2014). Marked corrugation and widening of elastic fibers pointed to disrupted structural integrity and elasticity, potentially increasing arterial stiffness. Enlarged and misshapen smooth muscle cell nuclei in the tunica media suggested cellular stress and aberrant remodeling (Laurent et al., 2001; Wagenseil and Mecham, 2009). Beyond direct arterial wall damage, CIS induced massive extravasated red blood cells beneath the tunica adventitia, indicative of microhemorrhages and increased vascular permeability. Fat necrosis of perivascular adipose tissue, with calcification and inflammatory cellular reaction, highlighted systemic toxicity extending beyond the vessel wall. Fibrinoid necrosis and additional calcification further underscored profound vascular pathology. Importantly, CAN completely prevented these histopathological alterations, demonstrating its potent ability to preserve aortic architecture and integrity. This comprehensive structural protection suggests the capacity of CAN to maintain endothelial barrier function, prevent pro-thrombotic events, inhibit vascular calcification, and preserve arterial elasticity. These effects are consistent with the known roles of CAN in attenuating ROS, inhibiting inflammatory signaling, and favorably modulating RAS signaling (Chen et al., 2008; Qie et al., 2020; Atwa et al., 2022).

Our results unequivocally demonstrate that CIS induces oxidative stress in the rat aorta, a key pathological mechanism underlying its vascular toxicity. This was evidenced by increased MDA, elevated MPO activity indicating oxidative burst and inflammatory cell infiltration (Zhang et al., 2002; Davies and Hawkins, 2020; Asfandiyar et al., 2024), and increased nitrite levels suggesting an imbalance in NO bioavailability, potentially forming peroxynitrite and contributing to oxidative/nitrosative stress (Radi, 2013). LPO alters membrane stability by compromising permeability and impairing membrane proteins, leading to impaired membrane function (Smathers et al., 2011). Concomitantly, CIS significantly depleted endogenous antioxidant defenses, including GSH and decreased activities of SOD, GPx, and GST. This renders vascular tissue highly vulnerable to oxidative damage. Elevated ROS induced by CIS can lead to detrimental effects, including LPO and membrane damage, oxidation of cellular proteins and DNA, and mitochondrial dysfunction, ultimately promoting cell death (Asehnoune et al., 2004). Besides oxidative damage, ROS can activate a pro-inflammatory response via activation of TLR-4/NF-κB signaling (Asehnoune et al., 2004). Accordingly, we observed increased expression of TLR-4 and NF-κB, and increased levels of pro-inflammatory cytokines. Consistent with this, we found upregulated iNOS, which produces NO in inflammatory conditions, contributing to oxidative/nitrosative stress. Furthermore, increased pro-inflammatory cytokines (TNF-α and IL-1β) and decreased anti-inflammatory IL-10 indicated a profound pro-inflammatory shift in the aortic microenvironment. This cytokine imbalance perpetuates inflammation and cellular damage. TNF-α and IL-1β are master pro-inflammatory mediators capable of inducing endothelial activation, leukocyte recruitment, adhesion molecule expression, and promoting further ROS generation and cell death pathways (Ramesh and Reeves, 2002; Pober and Sessa, 2007).

CAN effectively prevented all these CIS-induced alterations. This suggests CAN exerts potent antioxidant effects, either by directly scavenging ROS, enhancing endogenous antioxidant enzyme activity, or preserving non-enzymatic antioxidants. Its ability to restore GSH levels and the activities of SOD, GPx, and GST is particularly significant, as these are primary defenses against oxidative damage. This antioxidant action is crucial for protecting vascular cells from LPO, protein oxidation, and DNA damage, preserving cellular integrity and function. In addition, CAN effectively suppressed the entire inflammatory cascade, alleviating TLR-4, NF-κB, iNOS, TNF-α, IL-1β, and IL-10 levels. This potent anti-inflammatory action, likely mediated primarily through AT1R blockade preventing Ang II-induced NF-κB activation, is crucial for interrupting the self-perpetuating cycle of inflammation central to CIS-induced vascular damage. This anti-inflammatory action aligns with previous studies highlighting its ability to modulate inflammatory pathways independently of AT1 receptor blockade, potentially via antioxidant effects or direct interaction with inflammatory mediators (Chen et al., 2008; Qie et al., 2020; Atwa et al., 2022).

This study introduces novel insights into necroptosis, a programmed necrotic cell death, in CIS-induced vascular toxicity. We observed increased levels of key necroptotic markers RIP1, RIP3, and MLKL in CIS-treated rat aorta. RIP1 and RIP3 form the necrosome, which activates MLKL, leading to cell lysis and DAMP release (Wang et al., 2014; Kearney and Martin, 2017). This indicates that necroptosis significantly contributes to CIS-induced vascular cell death. Interestingly, increased caspase-8, typically apoptotic, suggests a complex interplay between apoptotic and necroptotic pathways, as caspase-8 can regulate necroptosis (Dho et al., 2025). Necroptosis represents a lytic, pro-inflammatory form of programmed cell death, distinct from apoptosis. Its occurrence in the vasculature would lead to the release of intracellular DAMPs, amplifying local inflammation and tissue damage (Wu et al., 2024), a process consistent with the observed histopathological alterations and inflammatory cytokine surge. The ability of CAN to prevent the upregulation of RIP1, RIP3, MLKL, and caspase-8 provides compelling evidence that it effectively inhibits the necroptotic pathway. This represents a crucial mechanism contributing to its vasculoprotection, preventing the lytic demise of endothelial and vascular smooth muscle cells and mitigating DAMP-driven inflammation. Its ability to suppress necroptosis is significant, as necroptosis is a highly inflammatory cell death that exacerbates tissue injury and inflammation (Wang et al., 2014; Kearney and Martin, 2017). By inhibiting this pathway, CAN not only protects vascular cells from direct demise but also reduces pro-inflammatory mediator release. While our study focused on necroptosis markers, the observed reduction in IL-1β also hints at potential inhibition of other lytic pathways like pyroptosis.

To further explore the antioxidant, cytoprotective, and anti-inflammatory effects of CAN, we determined changes in the SIRT1/Nrf2/HO-1 signaling in rat aorta. CIS downregulated key components of the SIRT1/Nrf2/HO-1 signaling in rat aorta. SIRT1 is crucial for cellular stress responses, metabolism, and longevity, protecting against oxidative stress and inflammation (Haigis and Guarente, 2006; Yang et al., 2022). Nrf2 is a master regulator of antioxidant and detoxifying enzyme expression, including HO-1 and GCLC (Satta et al., 2017). HO-1 degrades heme into potent antioxidant and cytoprotective molecules (Pae and Chung, 2009). GCLC is the rate-limiting enzyme in GSH synthesis, explaining GSH depletion in CIS-treated rats (Lu, 2013). CAN effectively prevented CIS-induced downregulation of SIRT1, Nrf2, HO-1, and GCLC, and upregulation of Keap1. This indicates that CAN actively upregulates this crucial endogenous defense pathway, enhancing cellular capacity to combat oxidative stress and inflammation. SIRT1 activation by CAN can lead to Nrf2 deacetylation and activation, promoting HO-1 and GCLC transcription, and increasing antioxidant/cytoprotective molecule production. This synergistic activation of the SIRT1/Nrf2/HO-1 axis by CAN represents a powerful mechanism for its vasculoprotective effects. Molecular docking studies provide valuable insights into CAN’s potential molecular mechanisms, revealing binding affinity towards SIRT1, Keap1, and HO-1. This observation offers a potential mechanism for its observed upregulation of these cytoprotective proteins. Predicted binding to SIRT1 suggests direct interaction, explaining the ability of CAN to upregulate SIRT1 expression/activity and downstream protective pathways. Interaction with Keap1 could disrupt the Keap1-Nrf2 complex, facilitating Nrf2 release, nuclear translocation, and subsequent transcription of ARE-driven genes like HO-1 and GCLC (Satta et al., 2017). Binding to HO-1 suggests that CAN might not only induce HO-1 expression but also directly modulate its activity, contributing to antioxidant/cytoprotective effects. While these docking results require validation through functional assays and structural studies, they provide a plausible hypothesis for a direct, AT1R-independent mechanism by which CAN activates the SIRT1/Nrf2/HO-1 axis. This represents a potentially novel facet of CAN’s pharmacology relevant to cytoprotection.

The impact of CIS on the aorta included significant dysregulation of the local RAS, characterized by downregulated Ang-(1–7) and increased Ang II. Ang II primarily mediates vasoconstriction, inflammation, and fibrosis via the AT1 receptor, while Ang-(1–7) exerts vasodilatory, anti-inflammatory, and anti-fibrotic effects (Cau et al., 2021; Zhou et al., 2025). This imbalance, with elevated Ang II and reduced Ang-(1–7), indicates a shift towards a pro-hypertensive, pro-inflammatory, and pro-fibrotic state, contributing to CIS-induced vascular damage. CAN effectively prevented these CIS-induced alterations. By blocking the AT1 receptor, CAN directly counteracts Ang II’s detrimental effects, leading to vasodilation and reduced inflammation and fibrosis. AT1 receptor blockade can also indirectly increase Ang-(1–7) by shunting Ang I metabolism, enhancing its protective actions (Bihl et al., 2015). This dual RAS modulation suggests that CAN not only blocks the AT1R but may also promote a shift towards the protective RAS axis, potentially by reducing Ang II-mediated suppression of ACE2 or by other compensatory mechanisms. This rebalancing of the RAS towards the protective arm significantly contributes to the observed reduction in oxidative stress, inflammation, and structural damage.

The protective effect of CAN against CIS-induced vasculotoxicity was further supported by assessment of endothelium-dependent vasodilation. Myography studies revealed that CIS significantly decreased endothelium-dependent vasodilation in the rat aorta, indicating profound ED. This impairment suggests that CIS compromises endothelial NO production/release or smooth muscle cell responsiveness to NO. This endothelial dysfunction is a critical early event in cardiovascular disease, contributing to increased vascular resistance and hypertension (Sandoo et al., 2010). CAN effectively prevented this CIS-induced decrease, highlighting its ability to preserve/restore endothelial function. This beneficial effect likely stems from its multifaceted actions: reducing oxidative stress, suppressing inflammation, and modulating the RAS. By mitigating ROS and inflammatory mediators, CAN protects endothelial cells, preserving NO bioavailability and signaling. However, this study was limited to male healthy rats, and future work should include both sexes to enhance the generalizability of these findings. In addition, further studies using tumor-bearing models are warranted to determine whether the vascular protective effects of CAN are preserved in the setting of cancer. Another limitation is that systemic blood pressure was not measured. Although aortic endothelial function was assessed *ex vivo*, future studies should include *in vivo* hemodynamic monitoring to fully characterize the cardiovascular effects of CIS and the protective action of CAN.

## 5 Conclusion

This study provides comprehensive evidence that CAN effectively mitigates CIS-induced vascular toxicity in rats through a multi-pronged approach. The data collectively support a model where CIS inflicts vascular toxicity through a synergistic network of insults, including oxidative stress, TLR-4/NF-κB-driven inflammation, activation of lytic necroptosis, and a deleterious shift in the local RAS balance, culminating in the downregulation of the cytoprotective SIRT1/Nrf2/HO-1 axis. The protective effects of CAN encompass the preservation of aortic histoarchitecture, significant reduction of oxidative stress, potent suppression of inflammatory signaling and pro-inflammatory cytokines, inhibition of necroptotic cell death, restoration of RAS balance, and upregulation of SIRT1/Nrf2/HO-1 pathway. Functionally, CAN successfully restored endothelium-dependent vasodilation, highlighting its ability to preserve vascular function. The molecular docking insights further support these findings by suggesting direct interactions of CAN with key protective proteins like SIRT1, Keap1, and HO-1. These findings collectively establish CAN as a promising therapeutic agent for preventing CIS-induced vascular toxicity, warranting further investigation into its clinical application in cancer patients undergoing CIS chemotherapy.

## Data Availability

The manuscript contains all data supporting the reported results.
